# Bis(4-methyl­imidazolium) succinate succinic acid solvate

**DOI:** 10.1107/S1600536809006205

**Published:** 2009-02-25

**Authors:** Guihuan Du, Zuli Liu, Qian Chu, Zhen Li, Suming Zhang

**Affiliations:** a Department of Physics, Huazhong University of Science and Technology, Wuhan 430074, People’s Republic of China; b Tongji Hospital, Huazhong University of Science and Technology, Wuhan 430070, People’s Republic of China

## Abstract

In the title compound, 2C_4_H_7_N_2_^+^·C_4_H_4_O_4_^2−^·C_4_H_6_O_4_, the asymmetric unit consists of two 4-methyl­imidazolium cations, one succinate dianion and one netrual succinic acid mol­ecule and within the latter components, the C—O, C=O and C O bonds are clearly evidenced from their relative distances. In the crystal structure, the individual components are linked by inter­molecular N—H⋯O, O—H⋯O and C—H⋯O hydrogen bonds into a two-dimensional network parallel to the (101) plane in which *R*_3_^3^(9), *R*_3_^3^(12) and *R*_4_^4^(18) hydrogen-bond motifs are present.

## Related literature

For general background on co-crystals, see: Aakeröy & Salmon (2005 ▶); Aakeröy *et al.* (2007 ▶); Childs & Hardcastle (2007 ▶); Childs *et al.* (2007 ▶). For hydrogen-bond motifs, see: Bernstein *et al.* (1995 ▶).
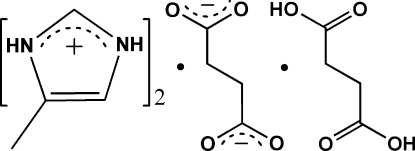


## Experimental

### 

#### Crystal data

2C_4_H_7_N_2_^+^·C_4_H_4_O_4_^2−^·C_4_H_6_O_4_
*M* *_r_* = 400.39
Monoclinic, 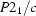

*a* = 17.260 (5) Å
*b* = 14.066 (4) Å
*c* = 7.761 (2) Å
β = 95.008 (6)°
*V* = 1877.0 (9) Å^3^
*Z* = 4
Mo *K*α radiation
μ = 0.12 mm^−1^
*T* = 296 K
0.30 × 0.10 × 0.04 mm

#### Data collection

Bruker SMART APEX CCD area-detector diffractometer
Absorption correction: multi-scan (*SADABS*; Sheldrick, 1996 ▶) *T*_min_ = 0.957, *T*_max_ = 0.995
20337 measured reflections
4080 independent reflections
2197 reflections with *I* > 2σ(*I*)
*R*_int_ = 0.063

#### Refinement

*R*[*F* ^2^ > 2σ(*F* ^2^)] = 0.056
*wR*(*F* ^2^) = 0.153
*S* = 0.95
4080 reflections
273 parameters
H atoms treated by a mixture of independent and constrained refinement
Δρ_max_ = 0.28 e Å^−3^
Δρ_min_ = −0.25 e Å^−3^

### 

Data collection: *SMART* (Bruker, 2001 ▶); cell refinement: *SAINT-Plus* (Bruker, 2001 ▶); data reduction: *SAINT-Plus*; program(s) used to solve structure: *SHELXS97* (Sheldrick, 2008 ▶); program(s) used to refine structure: *SHELXL97* (Sheldrick, 2008 ▶); molecular graphics: *PLATON* (Spek, 2009 ▶); software used to prepare material for publication: *PLATON*.

## Supplementary Material

Crystal structure: contains datablocks global, I. DOI: 10.1107/S1600536809006205/lh2776sup1.cif

Structure factors: contains datablocks I. DOI: 10.1107/S1600536809006205/lh2776Isup2.hkl

Additional supplementary materials:  crystallographic information; 3D view; checkCIF report

## Figures and Tables

**Table 1:** Selected bond lengths (Å)

C9—O1	1.235 (2)
C9—O2	1.271 (2)
C12—O4	1.228 (3)
C12—O3	1.276 (2)
C13—O6	1.210 (2)
C13—O5	1.298 (3)
C16—O7	1.207 (2)
C16—O8	1.302 (3)

**Table 2:** Hydrogen-bond geometry (Å, °)

*D*—H⋯*A*	*D*—H	H⋯*A*	*D*⋯*A*	*D*—H⋯*A*
N1—H1⋯O4^i^	0.96 (2)	1.74 (2)	2.699 (3)	176 (2)
N2—H2*A*⋯O3	0.97 (2)	1.78 (2)	2.752 (2)	173.0 (19)
N3—H3*A*⋯O1	1.07 (2)	1.61 (2)	2.673 (2)	170.6 (19)
N4—H4⋯O2^ii^	0.98 (2)	1.77 (2)	2.745 (2)	178.8 (19)
O5—H5⋯O3	0.98 (3)	1.53 (3)	2.509 (2)	177 (3)
O8—H8⋯O2^iii^	1.02 (3)	1.50 (3)	2.518 (2)	176 (3)
C2—H2⋯O6	0.93	2.29	3.024 (3)	136
C3—H3⋯O8^iv^	0.93	2.43	3.354 (3)	176
C6—H6⋯O5	0.93	2.43	3.346 (3)	169
C7—H7⋯O7^v^	0.93	2.29	3.017 (3)	134

Symmetry codes: (i) 
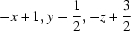
; (ii) 
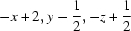
; (iii) 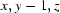; (iv) 
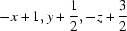
; (v) 
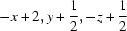
.

**Fig. 1.**
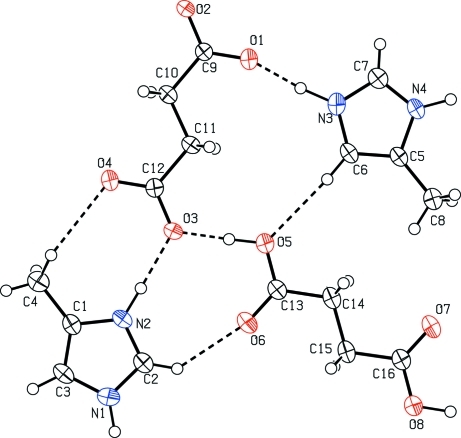
The asymmetric unit of (I), showing the atom-numbering scheme. Displacement ellipsoids are drawn at the 50% probability level. H-bonds are shown in dashed lines.

**Fig. 2.**
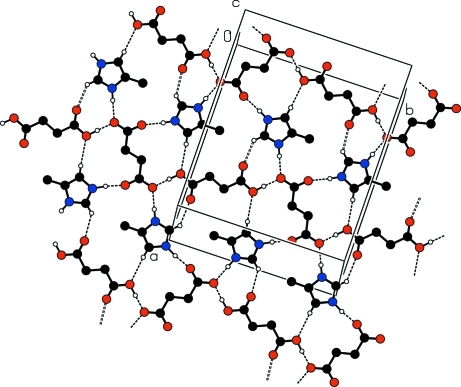
Part of the crystal structure of (I), showing the formation of the two-dimensional network parallel to the (101) plane linked by N-H···O, O-H···O and C-H···O hydrogen-bonds. Hydrogen atoms not involved in the motif have been omitted for clarity.

## supplementary crystallographic information

### Comment

In recent years, research on co-crystal or organic salts has been expanded rapidly owing to their potential application in the preparation of active pharmaceutical ingredients (Aakeröy *et al.*, 2007; Childs & Hardcastle, 2007; Childs *et al.*, 2007). In this paper, we report an organic salt formed by 4-methyl-imidazole and succinic acid in 95% methanol solution at room temperature, namely bis(4-methyl-imidazolium) succinicate succinic acid, (I).

In (I), the asymmetric unit is composed of two 4-methylimidazolium cations, one succinicate dianion and one netrual succinic acid molecule. The title compound can be regarded as an organic salt according to the definition of Aakeröy and Salmon (2005). One of the succinnic acid molecules is dually deprotonated, leading to a dianion (Fig. 1) which can be evidenced to an extent by the variations of the carboxyl C-O, C ═O and C ≐O bond distances (Table 1).

In the crystal structure, by a combination of four N-H···O and two O-H···O hydrogen bonds (Table 2) molecules in (I) are linked into a two-dimensional network parallel to the (101) plane (Fig.2) in which  *R*_3_^3^(9), *R*_3_^3^(12) and *R*_4_^4^(18) hydrogen-bonding motifs are present (Bernstein *et al.*,  1995). Within the network, several weak C-H..O interactions are present. No other interactions, such as C-H···π or π···π are observed in (I).

### Experimental

All the reagents and solvents were used as obtained without further purification. A 1:2 molar amounts of succinic acid (0.2 mmol, 23.6 mg) and 4-methyl-imidazole (0.4 mmol, 32.8 mg) were dissolved in 95% methanol (20 ml). The mixture was stirred for half an hour at room temperature and then filtered. The resulting solution was kept in air for one week. Plate crystals of (I) suitable for single-crystal X-ray diffraction analysis were grown by slow evaporation of the solution at the bottom of the vessel.

### Refinement

H atoms bonded to C atoms were located in difference maps and subsequently treated as riding, with C–H = 0.93 Å (aromatic), 0.97Å (methylene), 0.96Å (methyl), *U*_iso_(H) = 1.2*U*_eq_( aromatic and methylene C) and 1.5*U*_eq_( methyl C). H atoms bonded to N and O atoms were also found in difference maps and their distances were refined freely (see Table 1 for the distances), and the *U*_iso_(H) values being set k times of their carrier atoms ( k = 1.2 for N and 1.5 for O atoms)

### Crystal data

**Table:**

2C_4_H_7_N_2_^+^·C_4_H_4_O_4_^2^^−^·C_4_H_6_O_4_	*F*(000) = 848
*M**_r_* = 400.39	*D*_x_ = 1.417 Mg m^−^^3^
Monoclinic, *P*2_1_/*c*	Mo *K*α radiation, λ = 0.71073 Å
Hall symbol: -P 2ybc	Cell parameters from 2035 reflections
*a* = 17.260 (5) Å	θ = 2.4–21.1°
*b* = 14.066 (4) Å	µ = 0.12 mm^−^^1^
*c* = 7.761 (2) Å	*T* = 296 K
β = 95.008 (6)°	Plate, colorless
*V* = 1877.0 (9) Å^3^	0.30 × 0.10 × 0.04 mm
*Z* = 4	

### Data collection

**Table:**

Bruker SMART APEX CCD area-detector diffractometer	4080 independent reflections
Radiation source: fine focus sealed Siemens Mo tube	2197 reflections with *I* > 2σ(*I*)
graphite	*R*_int_ = 0.063
0.3° wide ω exposures scans	θ_max_ = 27.0°, θ_min_ = 1.9°
Absorption correction: multi-scan (*SADABS*; Sheldrick, 1996)	*h* = −22→22
*T*_min_ = 0.957, *T*_max_ = 0.995	*k* = −17→17
20337 measured reflections	*l* = −8→9

### Refinement

**Table:**

Refinement on *F*^2^	Primary atom site location: structure-invariant direct methods
Least-squares matrix: full	Secondary atom site location: difference Fourier map
*R*[*F*^2^ > 2σ(*F*^2^)] = 0.056	Hydrogen site location: inferred from neighbouring sites
*wR*(*F*^2^) = 0.153	H atoms treated by a mixture of independent and constrained refinement
*S* = 0.95	*w* = 1/[σ^2^(*F*_o_^2^) + (0.0817*P*)^2^] where *P* = (*F*_o_^2^ + 2*F*_c_^2^)/3
4080 reflections	(Δ/σ)_max_ < 0.001
273 parameters	Δρ_max_ = 0.28 e Å^−^^3^
0 restraints	Δρ_min_ = −0.25 e Å^−^^3^

### Special details

**Table:**

Geometry. All esds (except the esd in the dihedral angle between two l.s. planes) are estimated using the full covariance matrix. The cell esds are taken into account individually in the estimation of esds in distances, angles and torsion angles; correlations between esds in cell parameters are only used when they are defined by crystal symmetry. An approximate (isotropic) treatment of cell esds is used for estimating esds involving l.s. planes.
Refinement. Refinement of F^2^ against ALL reflections. The weighted R-factor wR and goodness of fit S are based on F^2^, conventional R-factors R are based on F, with F set to zero for negative F^2^. The threshold expression of F^2^ > 2sigma(F^2^) is used only for calculating R-factors(gt) etc. and is not relevant to the choice of reflections for refinement. R-factors based on F^2^ are statistically about twice as large as those based on F, and R- factors based on ALL data will be even larger.

### Fractional atomic coordinates and isotropic or equivalent isotropic displacement parameters (Å^2^)

**Table:**

	*x*	*y*	*z*	*U*_iso_*/*U*_eq_	
C1	0.45695 (11)	0.47579 (16)	0.7860 (3)	0.0375 (6)	
C2	0.50516 (13)	0.33293 (17)	0.7523 (3)	0.0476 (6)	
H2	0.5380	0.2839	0.7247	0.057*	
C3	0.40700 (12)	0.41101 (16)	0.8406 (3)	0.0431 (6)	
H3	0.3597	0.4241	0.8847	0.052*	
C4	0.45464 (13)	0.58059 (16)	0.7747 (3)	0.0498 (7)	
H4A	0.4367	0.5993	0.6590	0.075*	
H4B	0.5059	0.6057	0.8036	0.075*	
H4C	0.4199	0.6050	0.8540	0.075*	
C5	0.96099 (11)	0.34301 (16)	0.3015 (3)	0.0342 (5)	
C6	0.91078 (12)	0.40732 (16)	0.3567 (3)	0.0404 (6)	
H6	0.8665	0.3936	0.4122	0.049*	
C7	1.00090 (12)	0.48664 (17)	0.2388 (3)	0.0435 (6)	
H7	1.0302	0.5361	0.1986	0.052*	
C8	0.96426 (13)	0.23840 (16)	0.3093 (3)	0.0454 (6)	
H8A	0.9213	0.2150	0.3676	0.068*	
H8B	1.0122	0.2190	0.3714	0.068*	
H8C	0.9614	0.2131	0.1941	0.068*	
C9	0.83954 (12)	0.73296 (15)	0.4322 (3)	0.0327 (5)	
C10	0.76053 (11)	0.71074 (15)	0.4945 (3)	0.0352 (5)	
H10A	0.7558	0.7446	0.6020	0.042*	
H10B	0.7206	0.7348	0.4101	0.042*	
C11	0.74541 (12)	0.60712 (15)	0.5235 (3)	0.0377 (6)	
H11A	0.7865	0.5824	0.6046	0.045*	
H11B	0.7482	0.5737	0.4150	0.045*	
C12	0.66724 (12)	0.58515 (15)	0.5925 (3)	0.0374 (6)	
C13	0.70985 (12)	0.28540 (16)	0.5270 (3)	0.0380 (6)	
C14	0.76523 (12)	0.20813 (16)	0.4875 (3)	0.0398 (6)	
H14A	0.8133	0.2165	0.5604	0.048*	
H14B	0.7772	0.2145	0.3682	0.048*	
C15	0.73494 (12)	0.10900 (16)	0.5142 (3)	0.0449 (6)	
H15A	0.7241	0.1020	0.6341	0.054*	
H15B	0.6863	0.1009	0.4431	0.054*	
C16	0.79014 (13)	0.03232 (16)	0.4709 (3)	0.0410 (6)	
N1	0.43813 (11)	0.32252 (14)	0.8197 (3)	0.0457 (5)	
H1	0.4148 (14)	0.2622 (18)	0.842 (3)	0.055*	
N2	0.51776 (10)	0.42424 (13)	0.7306 (3)	0.0413 (5)	
H2A	0.5629 (13)	0.4491 (16)	0.678 (3)	0.050*	
N3	0.93627 (10)	0.49658 (14)	0.3170 (2)	0.0433 (5)	
H3A	0.9102 (13)	0.5635 (17)	0.342 (3)	0.052*	
N4	1.01708 (10)	0.39539 (13)	0.2269 (2)	0.0386 (5)	
H4	1.0622 (13)	0.3685 (15)	0.177 (3)	0.046*	
O1	0.88428 (9)	0.66845 (11)	0.3982 (2)	0.0565 (5)	
O2	0.85672 (8)	0.82023 (10)	0.4172 (2)	0.0453 (4)	
O3	0.65044 (8)	0.49741 (10)	0.6087 (2)	0.0461 (4)	
O4	0.62385 (9)	0.64950 (11)	0.6310 (3)	0.0647 (6)	
O5	0.73726 (9)	0.37014 (12)	0.5060 (2)	0.0542 (5)	
H5	0.7020 (17)	0.419 (2)	0.544 (3)	0.081*	
O6	0.64637 (10)	0.27081 (12)	0.5766 (3)	0.0671 (6)	
O7	0.84928 (10)	0.04663 (12)	0.4027 (3)	0.0639 (6)	
O8	0.76793 (10)	−0.05217 (12)	0.5142 (3)	0.0605 (6)	
H8	0.8020 (17)	−0.105 (2)	0.473 (4)	0.091*	

### Atomic displacement parameters (Å^2^)

**Table:**

	*U*^11^	*U*^22^	*U*^33^	*U*^12^	*U*^13^	*U*^23^
C1	0.0270 (11)	0.0344 (13)	0.0528 (14)	0.0012 (9)	0.0138 (10)	−0.0009 (11)
C2	0.0344 (13)	0.0358 (15)	0.0763 (18)	−0.0018 (11)	0.0253 (12)	−0.0020 (13)
C3	0.0284 (12)	0.0410 (15)	0.0625 (16)	−0.0039 (11)	0.0202 (11)	0.0011 (12)
C4	0.0381 (14)	0.0369 (15)	0.0772 (18)	−0.0010 (11)	0.0215 (13)	0.0039 (13)
C5	0.0255 (10)	0.0325 (13)	0.0465 (14)	−0.0003 (9)	0.0148 (10)	−0.0016 (10)
C6	0.0282 (12)	0.0356 (14)	0.0606 (16)	0.0007 (10)	0.0217 (11)	−0.0004 (11)
C7	0.0356 (13)	0.0347 (15)	0.0629 (16)	−0.0005 (10)	0.0199 (11)	−0.0009 (12)
C8	0.0384 (13)	0.0350 (14)	0.0663 (17)	0.0041 (10)	0.0233 (12)	0.0009 (12)
C9	0.0264 (11)	0.0247 (12)	0.0488 (14)	−0.0004 (9)	0.0130 (10)	−0.0008 (10)
C10	0.0279 (11)	0.0303 (13)	0.0497 (14)	−0.0003 (9)	0.0167 (10)	0.0027 (10)
C11	0.0284 (11)	0.0291 (13)	0.0583 (15)	−0.0022 (9)	0.0186 (11)	0.0014 (11)
C12	0.0307 (12)	0.0264 (13)	0.0573 (16)	0.0006 (10)	0.0162 (11)	−0.0019 (11)
C13	0.0300 (11)	0.0330 (14)	0.0532 (15)	0.0001 (10)	0.0164 (10)	0.0005 (11)
C14	0.0293 (12)	0.0372 (14)	0.0553 (15)	0.0034 (10)	0.0167 (11)	−0.0011 (11)
C15	0.0314 (12)	0.0370 (15)	0.0693 (17)	0.0051 (10)	0.0222 (12)	−0.0016 (12)
C16	0.0322 (12)	0.0341 (14)	0.0594 (15)	0.0004 (10)	0.0184 (11)	−0.0030 (11)
N1	0.0371 (11)	0.0327 (12)	0.0697 (14)	−0.0074 (9)	0.0195 (10)	0.0020 (10)
N2	0.0298 (10)	0.0333 (12)	0.0639 (13)	−0.0044 (8)	0.0223 (9)	0.0000 (10)
N3	0.0353 (10)	0.0325 (12)	0.0645 (14)	0.0056 (9)	0.0183 (9)	−0.0042 (10)
N4	0.0280 (10)	0.0344 (12)	0.0561 (13)	0.0016 (8)	0.0200 (9)	−0.0018 (9)
O1	0.0422 (10)	0.0304 (10)	0.1026 (14)	0.0040 (7)	0.0383 (9)	−0.0027 (9)
O2	0.0326 (9)	0.0257 (9)	0.0821 (12)	−0.0009 (7)	0.0309 (8)	0.0033 (8)
O3	0.0342 (8)	0.0266 (9)	0.0819 (12)	−0.0027 (7)	0.0300 (8)	0.0026 (8)
O4	0.0474 (10)	0.0309 (10)	0.1235 (16)	0.0039 (8)	0.0513 (11)	−0.0015 (10)
O5	0.0409 (9)	0.0291 (10)	0.0980 (15)	0.0005 (7)	0.0368 (10)	−0.0032 (9)
O6	0.0406 (10)	0.0420 (11)	0.1255 (16)	0.0021 (8)	0.0465 (11)	0.0062 (10)
O7	0.0452 (10)	0.0430 (11)	0.1108 (15)	0.0044 (8)	0.0480 (10)	0.0016 (10)
O8	0.0464 (10)	0.0310 (11)	0.1109 (16)	0.0043 (8)	0.0453 (10)	0.0041 (10)

### Geometric parameters (Å, °)

**Table:**

C1—C3	1.348 (3)	C10—C11	1.501 (3)
C1—N2	1.375 (3)	C10—H10A	0.9700
C1—C4	1.477 (3)	C10—H10B	0.9700
C2—N2	1.316 (3)	C11—C12	1.526 (3)
C2—N1	1.319 (3)	C11—H11A	0.9700
C2—H2	0.9300	C11—H11B	0.9700
C3—N1	1.371 (3)	C12—O4	1.228 (3)
C3—H3	0.9300	C12—O3	1.276 (2)
C4—H4A	0.9600	C13—O6	1.210 (2)
C4—H4B	0.9600	C13—O5	1.298 (3)
C4—H4C	0.9600	C13—C14	1.497 (3)
C5—C6	1.348 (3)	C14—C15	1.510 (3)
C5—N4	1.383 (3)	C14—H14A	0.9700
C5—C8	1.474 (3)	C14—H14B	0.9700
C6—N3	1.374 (3)	C15—C16	1.497 (3)
C6—H6	0.9300	C15—H15A	0.9700
C7—N4	1.319 (3)	C15—H15B	0.9700
C7—N3	1.323 (3)	C16—O7	1.207 (2)
C7—H7	0.9300	C16—O8	1.302 (3)
C8—H8A	0.9600	N1—H1	0.96 (2)
C8—H8B	0.9600	N2—H2A	0.97 (2)
C8—H8C	0.9600	N3—H3A	1.07 (2)
C9—O1	1.235 (2)	N4—H4	0.98 (2)
C9—O2	1.271 (2)	O5—H5	0.98 (3)
C9—C10	1.519 (3)	O8—H8	1.02 (3)
			
C3—C1—N2	105.6 (2)	C10—C11—H11A	108.6
C3—C1—C4	132.7 (2)	C12—C11—H11A	108.6
N2—C1—C4	121.66 (19)	C10—C11—H11B	108.6
N2—C2—N1	108.6 (2)	C12—C11—H11B	108.6
N2—C2—H2	125.7	H11A—C11—H11B	107.5
N1—C2—H2	125.7	O4—C12—O3	122.69 (19)
C1—C3—N1	107.98 (19)	O4—C12—C11	120.84 (19)
C1—C3—H3	126.0	O3—C12—C11	116.47 (18)
N1—C3—H3	126.0	O6—C13—O5	123.1 (2)
C1—C4—H4A	109.5	O6—C13—C14	123.7 (2)
C1—C4—H4B	109.5	O5—C13—C14	113.25 (18)
H4A—C4—H4B	109.5	C13—C14—C15	114.05 (17)
C1—C4—H4C	109.5	C13—C14—H14A	108.7
H4A—C4—H4C	109.5	C15—C14—H14A	108.7
H4B—C4—H4C	109.5	C13—C14—H14B	108.7
C6—C5—N4	105.58 (19)	C15—C14—H14B	108.7
C6—C5—C8	132.85 (19)	H14A—C14—H14B	107.6
N4—C5—C8	121.57 (18)	C16—C15—C14	113.57 (18)
C5—C6—N3	108.32 (18)	C16—C15—H15A	108.9
C5—C6—H6	125.8	C14—C15—H15A	108.9
N3—C6—H6	125.8	C16—C15—H15B	108.9
N4—C7—N3	109.1 (2)	C14—C15—H15B	108.9
N4—C7—H7	125.4	H15A—C15—H15B	107.7
N3—C7—H7	125.4	O7—C16—O8	123.0 (2)
C5—C8—H8A	109.5	O7—C16—C15	123.9 (2)
C5—C8—H8B	109.5	O8—C16—C15	113.10 (19)
H8A—C8—H8B	109.5	C2—N1—C3	108.21 (19)
C5—C8—H8C	109.5	C2—N1—H1	124.3 (15)
H8A—C8—H8C	109.5	C3—N1—H1	127.4 (15)
H8B—C8—H8C	109.5	C2—N2—C1	109.59 (18)
O1—C9—O2	122.35 (18)	C2—N2—H2A	123.4 (13)
O1—C9—C10	120.83 (19)	C1—N2—H2A	126.9 (13)
O2—C9—C10	116.82 (18)	C7—N3—C6	107.79 (19)
C11—C10—C9	114.85 (17)	C7—N3—H3A	124.3 (12)
C11—C10—H10A	108.6	C6—N3—H3A	127.9 (12)
C9—C10—H10A	108.6	C7—N4—C5	109.20 (18)
C11—C10—H10B	108.6	C7—N4—H4	126.0 (13)
C9—C10—H10B	108.6	C5—N4—H4	124.8 (13)
H10A—C10—H10B	107.5	C13—O5—H5	111.4 (16)
C10—C11—C12	114.83 (17)	C16—O8—H8	113.4 (16)
			
N2—C1—C3—N1	0.7 (3)	C14—C15—C16—O7	−8.0 (4)
C4—C1—C3—N1	179.0 (3)	C14—C15—C16—O8	172.0 (2)
N4—C5—C6—N3	−0.3 (3)	N2—C2—N1—C3	0.3 (3)
C8—C5—C6—N3	179.4 (2)	C1—C3—N1—C2	−0.6 (3)
O1—C9—C10—C11	4.6 (3)	N1—C2—N2—C1	0.1 (3)
O2—C9—C10—C11	−175.4 (2)	C3—C1—N2—C2	−0.5 (3)
C9—C10—C11—C12	177.83 (19)	C4—C1—N2—C2	−179.0 (2)
C10—C11—C12—O4	−4.7 (3)	N4—C7—N3—C6	0.2 (3)
C10—C11—C12—O3	175.8 (2)	C5—C6—N3—C7	0.0 (3)
O6—C13—C14—C15	0.2 (4)	N3—C7—N4—C5	−0.4 (3)
O5—C13—C14—C15	179.0 (2)	C6—C5—N4—C7	0.4 (3)
C13—C14—C15—C16	178.83 (19)	C8—C5—N4—C7	−179.3 (2)

### Hydrogen-bond geometry (Å, °)

**Table:**

*D*—H···*A*	*D*—H	H···*A*	*D*···*A*	*D*—H···*A*
N1—H1···O4^i^	0.96 (2)	1.74 (2)	2.699 (3)	176 (2)
N2—H2A···O3	0.97 (2)	1.78 (2)	2.752 (2)	173.0 (19)
N3—H3A···O1	1.07 (2)	1.61 (2)	2.673 (2)	170.6 (19)
N4—H4···O2^ii^	0.98 (2)	1.77 (2)	2.745 (2)	178.8 (19)
O5—H5···O3	0.98 (3)	1.53 (3)	2.509 (2)	177 (3)
O8—H8···O2^iii^	1.02 (3)	1.50 (3)	2.518 (2)	176 (3)
C2—H2···O6	0.93	2.29	3.024 (3)	136
C3—H3···O8^iv^	0.93	2.43	3.354 (3)	176
C6—H6···O5	0.93	2.43	3.346 (3)	169
C7—H7···O7^v^	0.93	2.29	3.017 (3)	134

Symmetry codes: (i) −*x*+1, *y*−1/2, −*z*+3/2; (ii) −*x*+2, *y*−1/2, −*z*+1/2; (iii) *x*, *y*−1, *z*; (iv) −*x*+1, *y*+1/2, −*z*+3/2; (v) −*x*+2, *y*+1/2, −*z*+1/2.
